# Genetic Polymorphisms Involved in Folate Metabolism and Maternal Risk for Down Syndrome: A Meta-Analysis

**DOI:** 10.1155/2014/517504

**Published:** 2014-12-04

**Authors:** Daniella Balduino Victorino, Moacir Fernandes de Godoy, Eny Maria Goloni-Bertollo, Érika Cristina Pavarino

**Affiliations:** ^1^Unidade de Pesquisa em Genética e Biologia Molecular (UPGEM), Faculdade de Medicina de São José do Rio Preto (FAMERP), Avenida Brigadeiro Faria Lima No. 5416, Bloco U-6, 15090-000 São José do Rio Preto, SP, Brazil; ^2^Núcleo Transdisciplinar para Estudo do Caos e da Complexidade (NUTECC), Faculdade de Medicina de São José do Rio Preto (FAMERP), 15090-000 São José do Rio Preto, SP, Brazil

## Abstract

Inconclusive results of the association between genetic polymorphisms involved in folate metabolism and maternal risk for Down syndrome (DS) have been reported. Therefore, this meta-analysis was conducted. We searched electronic databases through May, 2014, for eligible studies. Pooled odds ratios with 95% confidence intervals were used to assess the strength of the association, which was estimated by fixed or random effects models. Heterogeneity among studies was evaluated using *Q*-test and *I* ^2^ statistic. Subgroup and sensitivity analyses were also conducted. Publication bias was estimated using Begg's and Egger's tests. A total of 17 case-controls studies were included. There was evidence for an association between the *MTRR* c.66A>G (rs1801394) polymorphism and maternal risk for DS. In the subgroup analysis, increased maternal risk for DS was found in Caucasians. Additionally, the polymorphic heterozygote *MTHFD1* 1958GA genotype was associated significantly with maternal risk for DS, when we limit the analysis by studies conformed to Hardy-Weinberg equilibrium. Finally, considering *MTR* c.2756A>G (rs1805087), *TC2* c.776C>G (rs1801198), and *CBS* c.844ins68, no significant associations have been found, neither in the overall analyses nor in the stratified analyses by ethnicity. In conclusion, our meta-analysis suggested that the *MTRR* c.66A>G (rs1801394) polymorphism and *MTHFD1* c.1958G>A (rs2236225) were associated with increased maternal risk for DS.

## 1. Introduction

Down Syndrome (DS) is the phenotypic manifestation of trisomy of human chromosome 21 and is the most common genetic disorder of intellectual disability, characterized by dysmorphic features that are usual to almost all affected individuals, including craniofacial abnormalities and hypotonia [1, 2]. As reported, the average prevalence is 1 in 660 [3] and, in the majority of DS cases (90%), the chromosomal nondisjunction event is of maternal origin, occurring mainly during meiosis I in the maturing oocyte [4].

In several studies, advanced maternal age at conception (35 years or older) has been associated with increased risk of DS births in various parts of the world [5–7]. However, several women younger than 35 years at conception have had DS children and are also found to be predisposed to early chromosomal nondisjunction [8–10]. In 1999, James et al. [11] were the first to suggest a role for the abnormal folate metabolism in chromosome 21 nondisjunction as elevated maternal risk for DS, independent of maternal age.

Methionine synthase (MTR), methionine synthase reductase (MTRR), transcobalamin 2 (TC2), cystathionine beta synthase (CBS), and methylenetetrahydrofolate dehydrogenase (MTHFD1) are very important enzymes involved in folate/homocysteine (Hcy) metabolism and play essential roles in synthesis and repair of DNA and methylation reactions [12]. The methylation of Hcy to methionine is catalyzed by MTR using cobalamin (vitamin B12) as a cofactor, in which the MTR may become inactivated due to the oxidation of cobalamin cofactor [10, 13, 14]. The transmembrane transport of cobalamin is mediated by cobalamin-transporting proteins, such as transcobalamin 2 (TC2) [15]. Regeneration of inactive form of MTR into its active form requires reductive methylation of vitamin B12 via a reaction catalyzed by MTRR in which S-adenosylmethionine (SAM) is used as a methyl donor [10, 13, 14].

Cystathionine *β*-synthase (CBS), an enzyme involved in the transsulfuration cycle, is responsible for metabolizing Hcy into cystathionine, a middle step in the synthesis of cysteine [16]. Additionally, methylenetetrahydrofolate dehydrogenase 1 (*MTHFD1*), a trifunctional nicotinamide adenine dinucleotide phosphate-dependent cytoplasmic enzyme, catalyzes the sequential interconversion of tetrahydrofolate (THF) into the corresponding 10-formyl-THF, 5,10-methenyl-THF, and 5,10-methylene-THF [17], which play an important role in* de novo* purine and pyrimidine biosynthesis and, thus, the synthesis of DNA [18].

Genetic polymorphisms in key enzymes of folate metabolism have been identified in the alteration of the levels of folate and Hcy [19], in the enzyme activity decrease, and also in the Hcy remethylation rate [14, 20]. Therefore, changes in folate levels may influence the DNA stability and integrity [21, 22] or affect the methylation patterns and, thus, predispose it to the development of DS [10, 22–24].

Considering the functional effects of the* MTR* c.2756A>G (rs1805087),* MTRR* c.66A>G (rs1801394),* TC2* c.776C>G (rs1801198),* CBS* c.844ins68, and* MTHFD1* c.1958G>A (rs2236225) polymorphisms, it is expected that these polymorphisms may be associated with the maternal DS risk and several studies have been carried out to determine this association. However, the results remain inconclusive. To explain these issues, we conducted a systematical review and a meta-analysis from all eligible studies, in order to provide more exact estimate of the association among* MTR* c.2756A>G (rs1805087),* MTRR* c.66A>G (rs1801394),* TC2* c.776C>G (rs1801198),* CBS* c.844ins68, and* MTHFD1* c.1958G>A (rs2236225) polymorphisms and the maternal risk for DS.

## 2. Methods

### 2.1. Search Strategy

A systematic review of literature was performed in PubMed, EMBASE, and Lilacs-Scielo databases (last search update, May 2014). The keywords and subject terms used were as follows: (Down syndrome or trisomy 21) and (methionine synthase or methione synthase reductase or transcobalamin or cystathionine beta synthase or methylenetetrahydrofolate dehydrogenase or MTR or MTRR or CBS or TC2 or TCII or MTHFD1 or MTHFD-1 or A2756G or A66G or C776G or 844ins68 or G1958A). The reference lists of the retrieved studies were also screened in order to identify extra articles on this same topic. This research only included papers published in English, Spanish, or Portuguese.

### 2.2. Inclusion and Exclusion Criteria

The following inclusion criteria were used: (a) case-control studies design; (b) association studies that evaluated the association between* MTR* c.2756A>G (rs1805087),* MTRR* c.66A>G (rs1801394),* TC2* c.776C>G (rs1801198),* CBS* c.844ins68, or* MTHFD1* c.1958G>A (rs2236225) polymorphisms and the maternal risk for DS in case mothers (DSM) and in a control group of mothers (CM); (c) DSM are considered mothers that gave birth to at least one child with DS, and the CM are mothers that have given birth to children without reported abnormalities; (d) studies with detailed genotype and allele frequencies of the DSM and CM or with sufficient data to calculate them; (e) for articles published by the same population resource or by the same research group, only the article with the largest sample size or most recent study was included in this meta-analysis. Studies with insufficient data, review articles, abstracts, editorials, comments, letters, case reports, and animal studies were excluded.

### 2.3. Data Extraction and Quality Assessment

The data were extracted by two reviewers independently. Another reviewer was required in order to resolve the differences between them. The information extracted from each study includes the following: the first author's name, the publication's year, country, ethnicity, demography characteristics, genotyping method, genotype and allele frequencies, and the number of DSM and CM.

### 2.4. Statistical Analysis

A chi-square test was used to estimate the Hardy-Weinberg equilibrium (HWE) among the control subjects. The maternal risk was evaluated through the following comparisons: (1) allelic model (polymorphic allele versus wild-type allele); (2) codominant models (heterozygous versus wild-type homozygous and polymorphic homozygous versus wild-type homozygous); (3) dominant model (heterozygotes and homozygotes for the polymorphic allele versus wild-type homozygous); (4) recessive model (polymorphic homozygous versus heterozygotes and homozygotes for the wild-type allele). Subgroup analyses based on different ethnic populations (Caucasian and Brazilian) were also performed. Additionally, sensitivity analysis was used in order to examine the results stability by omitting one study at a time.

The pooled OR was estimated using fixed-effects (FE) [42] and random-effects (RE) [43] models according to heterogeneity. Heterogeneity among studies was calculated using the Chi-square based* Q*-test [44]. The effect of heterogeneity was also quantified using *I* ^2^ statistic [45], which ranges between 0 and 100%. When an absence of heterogeneity between studies was detected, the Mantel-Haenszel method in a FE model was used. In contrast, when heterogeneity between studies was present, the DerSimonian and Laird method in a RE model was adopted. The associations were indicated as a pooled odds ratio (OR) and 95% confidence intervals (CI).

Publication bias was examined by funnel plot method, in which the standard error of log (OR) of each study was plotted against its log (OR). The asymmetry in funnel plot is detected when publication bias is present. Funnel plot asymmetry was also determined by Begg's test [46] and Egger's linear regression test [47]. *P* ≤ 0.05 was considered statistically significant. Data analyses were performed using the Cochrane systematic review software Review Manager 5.2, BioEstat 5.3, and StatsDirect 1.9.15.

## 3. Results

### 3.1. Quantitative Data Synthesis

**Figure 1:**
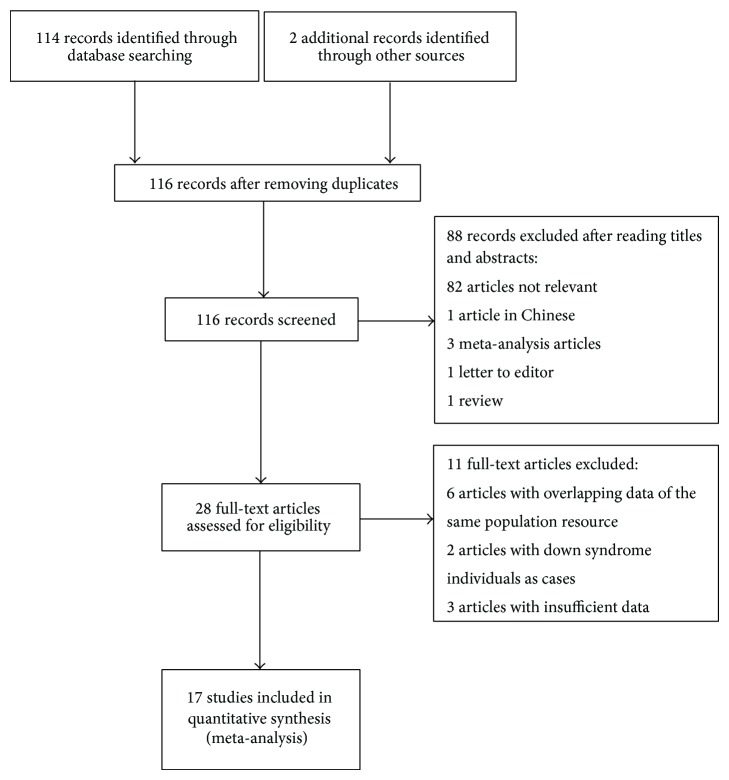
Flow diagram of eligible study selection process and studies excluded, with specification of reasons.

The literature search identified 116 potentially relevant studies; of these, 88 were excluded after screening the titles and abstracts. The full-text studies were retrieved for a detailed assessment. Eleven studies were excluded for specified reasons (6 articles with overlapping data of the same population resource, 2 articles with Down syndrome individuals as cases, and 3 articles with insufficient data). Finally, 17 case-control studies [25–41] with a total number of 1,988 DSM and 2,739 CM were included in the* MTR* c.2756A>G (rs1805087),* MTRR* c.66A>G (rs1801394),* TC2* c.776C>G (rs1801198),* CBS* c.844ins68, and* MTHFD1* c.1958G>A (rs2236225) meta-analysis (Figure 1).

Studies were conducted in different ethnic populations: seven involved Caucasian [25–27, 29, 35, 36, 40], seven Brazilian [28, 31–34, 37, 39], and three Asian [30, 38, 41]. Some of the articles reported that CM was composed of women who had no experience with miscarriages [25, 28, 30–36, 38–41], while other articles did not bring any information about miscarriages [26, 29]. On the other hand, two studies did report CM who had previous experiences with miscarriages [27, 37]. From the seventeen studies included in this meta-analysis, only two study reports of the parental origin of the extra chromosome 21 [31, 35]. The distribution of genotypes in the control groups of all the eligible studies was in agreement with HWE except for Chango et al. (*χ* ^2^ = 12.18, *P* = 0.0005) [27] and Ribeiro (*χ* ^2^ = 70.5, *P* < 0.0001) [32] in the* MTRR* c.66A>G (rs1801394), for Ribeiro (*χ* ^2^ = 4.14, *P* = 0.04) [32] and Liao et al. (*χ* ^2^ = 4.23, *P* = 0.03) [41] in the* TC2* c.776C>G (rs1801198), and for Scala et al. (*χ* ^2^ = 3.71, *P* = 0.05) [29] in the* MTHFD1* c.1958G>A (rs2236225) polymorphism. A list of the details extracted from the studies included in the meta-analysis is provided in Table 1.

### 3.2. Meta-Analyses, Test of Heterogeneity, Sensitivity, and Subgroup Analyses

#### 3.2.1. *MTR* c.2756A>G (rs1805087) Polymorphism and the Maternal Risk for DS

Firstly, we conducted meta-analysis of the effect of* MTR* c.2756A>G (rs1805087) polymorphism on the maternal risk for DS based on 8 case-control studies [27–29, 32, 33, 37, 39, 40] including 1,311 DSM and 1,674 CM. The results showed no significant association between all genetic models (Table 2). We then performed the subgroup analysis stratified by ethnicity. The pooled ORs from these analyses were also insignificant (Table 2).

#### 3.2.2. *MTRR* c.66A>G (rs1801394) Polymorphism and the Maternal Risk for DS

**Figure 2:**
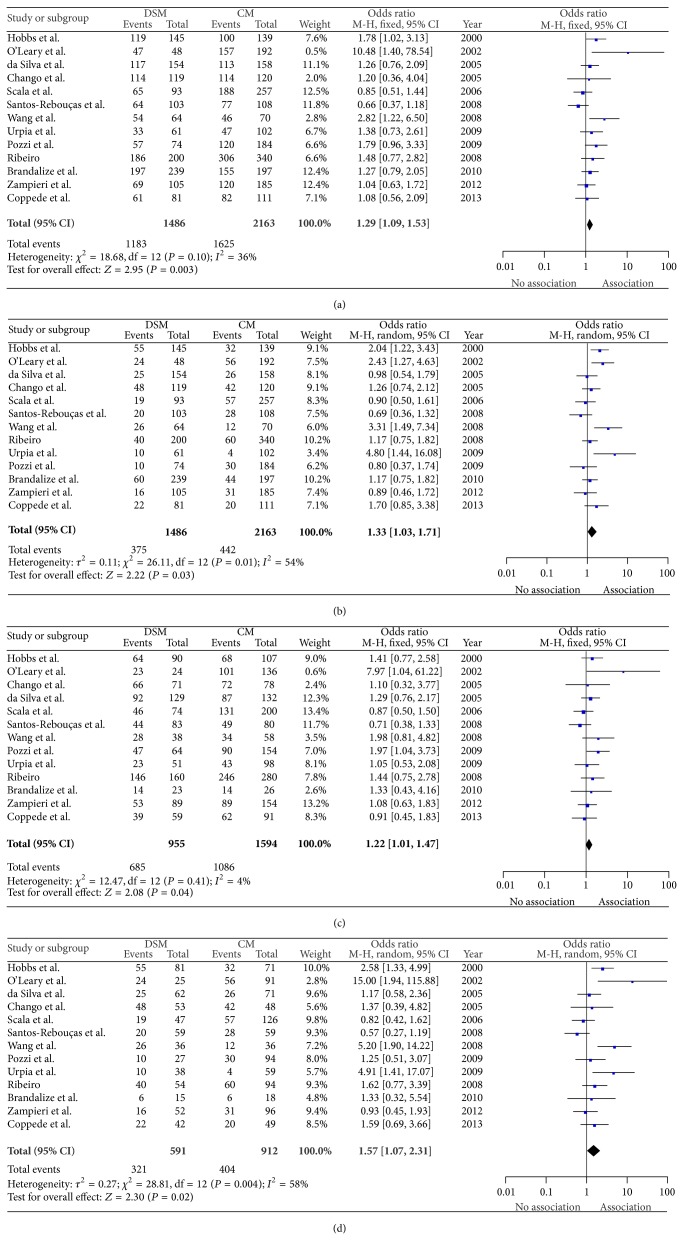
Forest plots showing the association between the* MTRR* c.66A>G (rs1801394) polymorphism and maternal risk for DS in overall population. GG/AG versus AA (a), GG versus AG/AA (b), AG versus AA (c), and GG versus AA (d) comparisons are illustrated. The squares represent odds ratios (ORs) and lines represent confidence intervals (95% CI). DSM: Down syndrome mothers; CM: control mothers.

We conducted meta-analysis based on 13 case-control studies [25–32, 34–37, 39], including 1,486 DSM and 2,163 CM. Overall, there was evidence for an association between the* MTRR* c.66A>G (rs1801394) polymorphism and maternal risk for DS in all genetic models (Table 2 and Figure 2), except in the allelic comparison (G versus A). However, there was significant heterogeneity among the studies (Table 2).

A reanalysis was carried out in order to exclude the studies whose control groups were not in HWE [27, 32] to assess the stability of the current analysis. The overall results did not change significantly after removing such studies, except to the AG versus AA comparison (OR = 1.20, 95% CI = 0.99 to 1.47) (Table 2). Additionally, the results showed that there still was heterogeneity among studies for the comparisons of GG/AG versus AA, GG versus AG + AA, and GG versus AA in all in HWE (Table 2). Subsequently, we performed subgroup analysis based on different ethnicities. Increased maternal risk for DS was observed in the Caucasian population (GG/AG versus AA: OR = 1.42, 95% CI = 1.08 to 1.88 and GG versus AG/AA: OR = 1.43, 95% CI = 1.13 to 1.83) (Table 2). No association was observed in any of the genetic models in Brazilians (Table 2). Subgroup analysis was not performed for* MTRR* c.66A>G (rs1801394) in Asians because there was only one study included [30]. For the subgroup analysis by Caucasians, no relevant changes in the results emerged from the exclusion of the study whose control group was not in HWE [27].

Sensitivity analysis was also performed and consisted of the analysis of every subgroup obtained by the exclusion of one single study at a time. It focused on checking the effect of each individual study, since the exclusion of a given article may isolate the remaining subgroup from the article's particular effect. None individual study significantly induced the heterogeneity among studies observed in the MTRR c.66A>G polymorphism analyses (data not shown). Additionally, the results indicated that no single study influenced the pooled OR qualitatively (data not shown). It suggested that the results of this meta-analysis were stable. For the subgroup analysis by Caucasians, after eliminating the results of O'Leary et al. [26] and Scala et al. [29], heterogeneity decreased, which indicated that these studies contribute to the heterogeneity in Caucasians. However, despite eliminating the data of these studies, our results did not change (data not show).

#### 3.2.3. *TC2* c.776C>G (rs1801198) Polymorphism and the Maternal Risk for DS

Four case-control studies [32, 33, 39, 41] with a total number of 495 DSM and 743 CM were included in this meta-analysis. No significant association between* TC2* c.776C>G (rs1801198) polymorphism and maternal risk for DS was found, neither for all populations nor for all populations in HWE (Table 2). Subgroup analysis was not performed due to the limited number of studies included.

#### 3.2.4. *C*β*S *c.844ins68 Polymorphism and the Maternal Risk for DS

We conducted meta-analysis of the effect of* C*β*S* c.844ins68 polymorphism on the maternal risk for DS based on 6 case-control studies [27–29, 33, 37, 39], including 825 DSM and 1,034 CM. As presented in Table 2, no significant association was found, neither when considering all populations nor for Brazilian population. Subgroup analysis was not performed in Caucasians because there were only two studies included.

#### 3.2.5. *MTHFD1 *c.1958G>A (rs2236225) Polymorphism and the Maternal Risk for DS

The association between* MTHFD1* c.1958G>A (rs2236225) polymorphism and maternal risk for DS was investigated in 5 studies [29, 32, 38, 39, 41] including 497 DSM and 930 CM. Overall, there was no significant association between* MTHFD1* c.1958G>A (rs2236225) polymorphism and maternal risk for DS when all populations are considered. However, the polymorphic heterozygote genotype GA was associated with significant maternal risk for DS (OR 1.33, 95% CI 1.01–1.75), compared with the wild-type homozygote genotype GG when we limit the analysis by HWE (Table 2). Subgroup analysis was not performed due to the limited number of studies included.

### 3.3. Publication Bias

The symmetry of funnel plots was examined visually by funnel plot and statistically by Begg's and [46] Egger's tests [47]. Appearances of the shapes of funnel plots seemed symmetrical in all comparisons. Additionally, Egger's tests also showed that there was no publication bias (*P* > 0.05) in all comparisons for all polymorphisms analyzed (Table 2).

## 4. Discussion

The present meta-analysis consists of an evaluation of* MTR* c.2756A>G (rs1805087),* MTRR* c.66A>G (rs1801394),* TC2* c.776C>G (rs1801198),* C*β*S* c.844ins68, and* MTHFD1* c.1958G>A (rs2236225) polymorphisms and maternal risk for DS. Our results show significant association between* MTRR* c.66A>G (rs1801394) polymorphism and maternal risk for DS in almost all genetic models when the general population is considered. Additionally, after the population was stratified by ethnicity, an increasing maternal risk for DS was observed in Caucasians. Furthermore, our results suggest that the* MTHFD1* 1958GA genotype is associated with maternal risk for DS and also that there is no significant association among* MTR* c.2756A>G (rs1805087),* TC2* c.776C>G (rs1801198), and* C*β*S* c.844ins68 polymorphisms and maternal risk for DS.

Folate is part of the B vitamins family and it is crucial for the synthesis of SAM, the major cellular methyl donor for DNA methylation [10, 48]. A common polymorphism reported in MTRR (66A → G) is the substitution of isoleucine by methionine on the residue 22. Such polymorphism changes the MTRR enzyme efficacy and also decreases the affinity of MTRR for MTR [14].

Based on the above, several studies were conducted in order to elucidate the association between the* MTRR* c.66A>G (rs1801394) polymorphism and maternal risk for DS. In our meta-analysis, we observed a significant association between the* MTRR* c.66A>G (rs1801394) polymorphism and maternal risk for DS in almost all genetic models, which corroborates with some of the previous case-control studies [25, 26, 30]. Although the exact mechanism is not yet determined, one possibility is related to the fact that the* MTRR* c.66A>G (rs1801394) polymorphism can lead to a decrease in the MTRR enzyme activity [14] and, since the MTRR enzyme converts the MTR enzyme from its inactive form to its active state [10, 13, 14], such a decrease may result in plasma Hcy elevation and DNA hypomethylation.

The DNA methylation is very important to the regulation of gene expression, to genomic integrity, and also to stability and chromatin organization [21, 22, 49, 50]. Several researchers have demonstrated that low folate status can affect the global methylation of DNA [51–54] and, thus, increase the frequency of chromosomal breaks [55], abnormal chromatin conformation, and DNA instability [56–59]. Such DNA instability may result in abnormal chromosome segregation [23, 60, 61] and consequently in aneuploidy [22–24, 48, 56, 57].

Since moderate heterogeneity is present in our meta-analysis, we decided to perform a stratified analyses based on HWE and on ethnicity. However, subgroup and sensitivity analyses were not able to find the source of heterogeneity. In our meta-analysis, we tried to minimize the heterogeneity between studies by performing a very careful search strategy and study selection. To accomplish that, we used an explicit inclusion criteria and performed quality data extraction and analysis. Despite all these efforts, a significant interstudy heterogeneity was present in some of the comparisons. It is necessary to point out that heterogeneity among studies is frequently observed in meta-analysis studies that report genetic associations [62], and, in the present meta-analysis, the observed heterogeneity may be due to ethnic variations, environmental interactions related to folate metabolism [63], and methodological reasons. Although the sources of heterogeneity cannot be easily detected [64, 65], the sensitivity analysis did not change the pooled results, which indicates that our results were statistically robust. Finally, an optional method available to investigate this problem is the meta-regression analysis [66]. Admittedly, one limitation of this method lies in the number of available studies with detailed covariates information, which prevents a more robust assessment of heterogeneity sources [67].

In our meta-analysis, the evidence suggested that* MTR* c.2756A>G (rs1805087),* TC2* c.776C>G (rs1801198), and* C*β*S* c.844ins68 polymorphisms did not contribute as an independent risk factor for DS. Our current data agrees with several previous performed case-control studies [27, 29, 33, 39, 41]. For the subgroup analysis based on ethnicity, we did not observe any effect modification. Some explanations might be responsible for the lack of association among* MTR* c.2756A>G (rs1805087),* TC2* c.776C>G (rs1801198), and* C*β*S* c.844ins68 polymorphisms and maternal risk for DS. First of all, the sample size of studies was relatively small. Secondly, risk factor may depend on genetic polymorphisms and potential gene-gene interaction. Several researchers showed that the polymorphisms are able to interact with each other and such interaction may modify their individual effects [28, 30, 37, 68]. Additionally, gene-environment interaction as the interaction between genotype and dietary intake, especially folate intake, may be decisive for maintaining the effects of these polymorphisms [63, 69].

To the best of our knowledge, our study was the first meta-analysis to investigate the association among* TC2* c.776C>G (rs1801198) and* MTHFD1* c.1958G>A (rs2236225) polymorphisms and maternal risk for DS. Our result suggests that the presence of the* MTHFD1* 1958GA genotype might be associated with maternal risk for DS. Previous studies have supported that* MTHFD1* c.1958G>A (rs2236225) polymorphism is able to reduce the activity and stability of the MTHFD1 enzyme and has been associated with an increased risk of neural tube defects [70] and unexplained second semester pregnancy loss [71]. Moreover, some studies reported that the combined* MTHFR* 677CT/TT and* MTHFD* 1958AA/GA [41] and* MTHFD* 1958AA/*RFC1* 80GG genotypes [29] were significantly associated with the maternal risk for DS. Since the number of included studies in* MTHFD1* c.1958G>A (rs2236225) meta-analysis was only 5, larger sample studies should be conducted in order to confirm this result.

To the best of our knowledge, there are three meta-analyses papers that reported the association between genetic polymorphisms involved in folate metabolism and maternal risk for DS [40, 72, 73]. Such meta-analyses reported distinct results and their included studies and sample sizes are different. There are some discrepancies between Yang's study [72] and our study. We performed some independent and original analyses, such as comparisons by genetic models. We demonstrated important results in those analyses, since the dominant, recessive, and codominant comparisons showed significant associations between* MTRR* c.66A>G (rs1801394) polymorphism and maternal risk for DS. Moreover, we were the first ones to conduct meta-analyses to evaluate the association among* TC2* c.776C>G (rs1801198) and* MTHFD1* c.1958G>A (rs2236225) polymorphisms and maternal risk for DS. Coppedè et al. [40] conducted another meta-analysis, performed almost simultaneously. In our study, the stratified analyses by codominant models were performed. However, such an analysis was not made in the Coppedè et al. [40] article. In addition, our meta-analysis included all the published studies and added another 363 DSM and 603 CM. Finally, the meta-analysis conducted by Amorim and Lima [73] only observed a significant association between G allele of the* MTRR* c.66A>G (rs1801394) polymorphism and maternal risk for DS. However, we observed important results for such polymorphism: the heterozygote AG and polymorphic homozygote GG genotypes, dominant and recessive genetic models, were significantly associated with maternal risk for DS. Their study only comprised eleven case-control studies, while our study included two additional articles, which accounted for 20% of the total sample size. We also conducted stratified analyses by ethnicity, which was not made in the Amorim and Lima [73] study. In conclusion, for the reasons illustrated above, we demonstrated stronger evidence and more powerful pooled results in comparison with previous meta-analyses.

There are still some limitations in this meta-analysis that need to be mentioned. Firstly, all included studies were performed as case-control studies, which prevent additional comments on a cause-effect relationship [74]. Secondly, since we only included studies written in English, Spanish, and Portuguese, a potential selection bias cannot be totally excluded. Thirdly, although previous reports have suggested that genetic polymorphisms involved in folate metabolism have a synergistic effect on enzyme activity [68, 75], we could not investigate the association between the combined genotypes of these polymorphisms and maternal risk for DS due to the lack of detailed original data in the included studies. In addition, such lack of data as folate intake and maternal age at conception, in the included studies, limited our further stratified analysis. Fourthly, we did not analyze gene-gene and gene-environment interactions. It is possible that specific environmental and lifestyle factors can influence the associations between genetic polymorphisms and maternal risk for DS.

In conclusion, our meta-analysis provided evidence that* MTRR* c.66A>G (rs1801394) polymorphism was associated with maternal risk for DS, especially in Caucasians. Additionally, our result suggested that the* MTHFD1* 1958GA genotype could be associated with maternal risk for DS. Finally, the evidence demonstrated that* MTR* c.2756A>G (rs1805087),* TC2* c.776C>G (rs1801198), and* C*β*S* c.844ins68 polymorphisms did not contribute as an independent risk factor of DS. Further larger and well-designed studies are required to confirm this conclusion; functional studies should also be conducted to fully understand the molecular mechanism of DS.

## Figures and Tables

**Table 1:** Characteristics of the included studies in meta-analysis.

First author	Year	Ethnicity	DSM^a^	CM^b^	Polymorphisms studied and included in meta-analysis	Genotype analysis
Hobbs et al. [25]	2000	Caucasian	145	139	*MTRR* c.66A>G	PCR/RFLP
O'Leary et al. [26]	2002	Caucasian	48	192	*MTRR* c.66A>G	PCR/RFLP
Chango et al. [27]	2005	Caucasian	119	120	*MTRR* c.66A>G/*MTR* c.2756A>G/*CBS* c.844ins68	PCR/RFLP
da Silva et al. [28]	2005	Brazilian	154	158	*MTRR* c.66A>G/*MTR* c.2756A>G/*CBS* c.844ins68	PCR/RFLP
Scala et al. [29]	2006	Caucasian	93	257	*MTRR* c.66A>G/*MTR* c.2756A>G/*CBS* c.844ins68/*MTHFD1 *c.1958G>A	PCR/RFLP
Wang et al. [30]	2008	Asian	64	70	*MTRR* c.66A>G	PCR/RFLP
Santos-Rebouças et al. [31]	2008	Brazilian	103	108	*MTRR* c.66A>G	PCR/RFLP
Ribeiro [32]	2008	Brazilian	200	340	*MTRR* c.66A>G/*MTR* c.2756A>G/*TC2 *c.776C>G/*MTHFD1 *c.1958G>A	PCR/RFLP
Fintelman-Rodrigues et al. [33]	2009	Brazilian	114	110	*MTR* c.2756A>G/*CBS* c.844ins68/*TC2 *c.776C>G	PCR/RFLP
Urpia [34]	2009	Brazilian	61	102	*MTRR* c.66A>G	PCR/RFLP
Pozzi et al. [35]	2009	Caucasian	74	184	*MTRR* c.66A>G	PCR/RFLP
Coppedè et al. [36]	2009	Caucasian	81	111	*MTRR* c.66A>G	PCR/RFLP
Brandalize et al. [37]	2010	Brazilian	239	197	*MTRR* c.66A>G/*MTR* c.2756A>G/*CBS* c.844ins68	PCR/RFLP
Neagos et al. [38]	2010	Asian	26	46	*MTHFD1 *c.1958G>A	PCR/RFLP
Zampieri et al. [39]	2012	Brazilian	105	185	*MTRR* c.66A>G/*MTR* c.2756A>G/*CBS* c.844ins68/*TC2 *c.776C>G/*MTHFD1 *c.1958G>A	PCR/RFLP
Coppedè et al. [40]	2013	Caucasian	286	305	*MTR* c.2756A>G	PCR/RFLP
Liao et al. [41]	2014	Asian	76	115	*TC2 *c.776C>G/*MTHFD1 *c.1958G>A	PCR/RFLP

^a^DSM: case mothers.

^b^CM: controls mothers.

**Table 2:** Pooled estimates and stratified analysis for the associations between *MTR* c.2756A>G (rs1805087), *MTRR* c.66A>G (rs1801394), *TC2* c.776C>G (rs1801198), *CBS* c.844ins68, and *MTHFD1* c.1958G>A (rs2236225) polymorphisms and maternal risk for Down syndrome.

Polymorphism	Comparison	Population	Study (*n*)	Test of association		Test of heterogeneity			Tests of publication bias (*P* value)	
				OR (95% CI)	*P* value (*Z* test)	*χ* ^2^	*I* ^2^	*P* value	Rank test (Begg and Mazumdar [46])	Linear regression (Egger et al. [47])
*MTR* c.2756A>G	GG/AG versus AA	All	8	1.14 (0.97–1.33)^b^	0.11	7.01	0%	0.43	0.27	0.20
		Caucasian	3	1.08 (0.84–1.40)^b^	0.55	2.69	26%	0.26	—	—
		Brazilian	5	1.17 (0.96–1.42)^b^	0.12	4.11	3%	0.39	0.48	0.65
	GG versus AG/AA	All	8	1.18 (0.80–1.76)^b^	0.41	3.69	0%	0.81	0.27	0.34
		Caucasian	3	0.80 (0.35–1.82)^b^	0.60	0.72	0%	0.70	—	—
		Brazilian	5	1.34 (0.85–2.12)^b^	0.21	1.92	0%	0.75	0.23	0.18
	AG versus AA	All	8	1.13 (0.96–1.33)^b^	0.15	9.26	24%	0.23	0.06	0.16
		Caucasian	3	1.10 (0.85–1.44)^b^	0.46	3.24	38%	0.20	—	—
		Brazilian	5	1.14 (0.93–1.40)^b^	0.21	5.99	33%	0.20	0.48	0.58
	GG versus AA	All	8	1.25 (0.84–1.88)^b^	0.27	2.97	0%	0.89	0.54	0.43
		Caucasian	3	0.85 (0.37–1.94)^b^	0.70	0.61	0%	0.74	—	—
		Brazilian	5	1.42 (0.89–2.27)^b^	0.14	1.28	0%	0.86	0.48	0.19
	G versus A	All	8	1.11 (0.97–1.26)^b^	0.14	5.33	0%	0.62	0.27	0.29
		Caucasian	3	1.04 (0.83–1.31)^b^	0.71	1.82	0%	0.40	—	—
		Brazilian	5	1.14 (0.96–1.34)^b^	0.12	3.18	0%	0.53	0.81	0.73

*MTRR* c.66A>G	GG/AG versus AA	All	13	**1.29 **(1.09–1.53)^b^	0.003	18.68	36%	0.10	0.25	0.08
		All in HWE	11	**1.28 **(1.00–1.65)^a^	0.05	18.42	46%	0.05	0.21	0.06
		Caucasian	6	**1.42 **(1.08–1.88)^b^	0.01	9.39	47%	0.09	0.46	0.31
		Brazilian	6	1.14 (0.91–1.42)^b^	0.25	4.87	0%	0.43	0.46	0.89
	GG versus AG/AA	All	13	**1.33 **(1.03–1.71)^a^	0.03	26.11	54%	0.01	0.59	0.27
		All in HWE	11	**1.37 **(1.00–1.88)^a^	0.05	25.82	61%	0.004	0.44	0.34
		Caucasian	6	**1.43 **(1.13–1.83)^b^	0.003	9.44	47%	0.09	0.46	0.64
		Brazilian	6	1.09 (0.86–1.37)^b^	0.47	8.35	40%	0.14	0.71	0.44
	AG versus AA	All	13	**1.22 **(1.01–1.47)^b^	0.04	12.47	4%	0.41	0.36	0.17
		All in HWE	11	1.20 (0.99–1.47)^b^	0.07	12.11	17%	0.28	0.44	0.13
		Caucasian	6	1.31 (0.98–1.75)^b^	0.07	7.89	37%	0.16	0.46	0.36
		Brazilian	6	1.11 (0.86–1.43)^b^	0.44	2.98	0%	0.70	0.46	0.60
	GG versus AA	All	13	**1.57 **(1.07–2.31)^a^	0.02	28.81	58%	0.004	0.30	0.07
		All in HWE	11	**1.61 **(1.02–2.53)^a^	0.04	28.72	65%	0.001	0.35	0.07
		Caucasian	6	1.65 (0.95–2.88)^a^	0.08	10.87	54%	0.05	>0.99	0.41
		Brazilian	6	1.16 (0.83–1.62)^b^	0.37	9.88	49%	0.08	>0.99	0.33
	G versus A	All	13	1.18 (0.99–1.40)^a^	0.07	35.50	66%	0.0004	0.20	0.12
		All in HWE	11	1.20 (0.96–1.49)^a^	0.10	35.38	72%	0.0001	0.28	0.12
		Caucasian	6	1.26 (0.96–1.66)^a^	0.10	13.85	64%	0.02	>0.99	0.84
		Brazilian	6	1.00 (0.88–1.14)^b^	0.97	8.26	39%	0.14	0.71	0.64

*C*β*S* c.844ins68	Ins +/+ + Ins −/+ versus Ins −/−	All	6	1.03 (0.80–1.31)^b^	0.84	2.60	0%	0.76	0.13	0.22
		Brazilian	4	1.10 (0.83–1.45)^b^	0.51	1.37	0%	0.71	0.75	0.79
	Ins +/+ versus Ins −/+ + Ins −/−	All	6	1.07 (0.50–2.28)^b^	0.86	2.96	0%	0.56	0.75	0.73
		Brazilian	4	1.17 (0.53–2.56)^b^	0.70	2.42	0%	0.49	0.75	0.75
	Ins −/+ versus Ins −/−	All	6	1.02 (0.79–1.32)^b^	0.87	3.33	0%	0.65	0.27	0.36
		Brazilian	4	1.09 (0.81–1.45)^b^	0.57	2.47	0%	0.48	0.75	0.99
	Ins +/+ versus Ins −/−	All	6	1.10 (0.51–2.34)^b^	0.81	2.69	0%	0.61	0.75	0.74
		Brazilian	4	1.20 (0.54–2.64)^b^	0.65	2.10	0%	0.55	0.33	0.76
	Ins + versus Ins −	All	6	1.07 (0.86–1.34)^b^	0.54	1.33	0%	0.93	0.71	0.65
		Brazilian	4	1.07 (0.83–1.37)^b^	0.61	0.51	0%	0.92	0.33	0.21

*MTHFD-1 *c.1958G>A	AA + GA versus GG	All	5	1.22 (0.96–1.55)^b^	0.10	1.86	0%	0.76	0.48	0.38
		All in HWE	4	1.28 (0.98–1.67)^b^	0.07	1.16	0%	0.76	0.33	0.21
	AA versus GA + GG	All	5	0.96 (0.71–1.30)^b^	0.79	1.61	0%	0.81	0.08	0.43
		All in HWE	4	0.96 (0.68–1.37)^b^	0.84	1.60	0%	0.66	0.33	0.53
	GA versus GG	All	5	1.26 (0.98–1.62)^b^	0.07	2.62	0%	0.62	0.48	0.37
		All in HWE	4	**1.33 **(1.01–1.75)^b^	0.04	1.86	0%	0.60	0.75	0.27
	AA versus GG	All	5	1.05 (0.74–1.50)^b^	0.77	0.66	0%	0.96	0.81	0.81
		All in HWE	4	1.09 (0.73–1.62)^b^	0.67	0.54	0%	0.91	0.75	0.85
	A versus G	All	5	1.08 (0.92–1.27)^b^	0.35	1.30	0%	0.86	0.48	0.40
		All in HWE	4	1.11 (0.93–1.33)^b^	0.26	0.87	0%	0.83	0.75	0.39

*TC2 *c.776C>G	GG + CG versus CC	All	4	1.27 (0.83–1.93)^a^	0.27	8.03	63%	0.05	0.33	0.53
		All in HWE	2	0.92 (0.64–1.32)^b^	0.66	0.38	0%	0.53	—	—
	GG versus CG + CC	All	4	0.94 (0.70–1.27)^b^	0.70	5.65	47%	0.13	0.75	0.91
		All in HWE	2	1.12 (0.40–3.16)^a^	0.83	4.32	77%	0.04	—	—
	CG versus CC	All	4	1.34 (0.82–2.19)^a^	0.25	9.74	69%	0.02	0.75	0.67
		All in HWE	2	0.89 (0.61–1.31)^b^	0.56	0	0%	0.96	—	—
	GG versus CC	All	4	1.22 (0.87–1.72)^b^	0.25	4.61	35%	0.20	0.75	0.57
		All in HWE	2	1.04 (0.61–1.78)^b^	0.88	3.60	72%	0.06	—	—
	G versus C	All	4	1.12 (0.95–1.32)^b^	0.19	6.17	51%	0.10	0.08	0.20
		All in HWE	2	0.97 (0.75–1.25)^b^	0.79	2.63	62%	0.10	—	—

OR: odds ratio; CI: confidence interval.

Bold values indicate significant associations.

^a^Random-effect model.

^b^Fixed-effect model.

—: insufficient strata.
